# Isolation and Characterization of scFv Antibody against Internal Ribosomal Entry Site of Enterovirus A71

**DOI:** 10.3390/ijms24129865

**Published:** 2023-06-07

**Authors:** Su Thandar Hlaing, Potjanee Srimanote, Pongsri Tongtawe, Onruedee Khantisitthiporn, Kittirat Glab-ampai, Monrat Chulanetra, Jeeraphong Thanongsaksrikul

**Affiliations:** 1 Graduate Program in Biomedical Sciences, Faculty of Allied Health Sciences, Thammasat University, Pathumtani 12120, Thailand; suthandar31@gmail.com (S.T.H.); psrimanote01@yahoo.com.au (P.S.); ptongtawe@ymail.com (P.T.); 2 Thammasat University Research Unit in Molecular Pathogenesis and Immunology of Infectious Diseases, Thammasat University, Pathumthani 12120, Thailand; yuleeko@hotmail.com; 3 Department of Medical Technology, Faculty of Allied Health Sciences, Thammasat University, Pathumthani 12120, Thailand; 4 Center of Research Excellence in Therapeutic Proteins and Antibody Engineering, Department of Parasitology, Faculty of Medicine Siriraj Hospital, Mahidol University, Bangkok 10700, Thailand; kittirat.gla@mahidol.edu (K.G.-a.); monchu82@gmail.com (M.C.)

**Keywords:** enterovirus, hand-foot-mouth disease, Enterovirus A71, internal ribosomal entry site, single-chain variable fragment

## Abstract

Enterovirus A71 (EV-A71) is one of the causative agents of hand-foot-mouth disease, which can be associated with neurocomplications of the central nervous system. A limited understanding of the virus’s biology and pathogenesis has led to the unavailability of effective anti-viral treatments. The EV-A71 RNA genome carries type I internal ribosomal entry site (IRES) at 5′ UTR that plays an essential role in the viral genomic translation. However, the detailed mechanism of IRES-mediated translation has not been elucidated. In this study, sequence analysis revealed that the domains IV, V, and VI of EV-A71 IRES contained the structurally conserved regions. The selected region was transcribed in vitro and labeled with biotin to use as an antigen for selecting the single-chain variable fragment (scFv) antibody from the naïve phage display library. The so-obtained scFv, namely, scFv #16-3, binds specifically to EV-A71 IRES. The molecular docking showed that the interaction between scFv #16-3 and EV-A71 IRES was mediated by the preferences of amino acid residues, including serine, tyrosine, glycine, lysine, and arginine on the antigen-binding sites contacted the nucleotides on the IRES domains IV and V. The so-produced scFv has the potential to develop as a structural biology tool to study the biology of the EV-A71 RNA genome.

## 1. Introduction

Enterovirus 71 (EV-A71) is one of the main etiological agents of hand-foot-mouth disease (HFMD). The disease has become a worldwide health-challenging issue since the 1960s [1]. HFMD epidemics have been more prominent in the Asia-Pacific region including Thailand, Japan, Hong Kong, Taiwan, and South Korea for several decades [2]. The cyclical epidemic of the disease happens every 2–3 years and sporadic cases are reported throughout the year in tropical regions such as Thailand, Vietnam, Singapore, and Malaysia. The common symptoms are fever with blisters in the hands and feet as well as in the mouth. Although HFMD is mostly self-limited, it sometimes develops complications with severe outcomes such as brainstem meningitis, encephalitis, and pulmonary edema, particularly in children younger than 5 years old. HFMD can be commonly transmitted through direct contact, ingestion of foods, and water contaminated with the patient’s nasopharyngeal secretions or fecal-oral route. The effective and specific treatment of the disease has not yet been established and this is probably due to the limited knowledge of the virus’s biology and its pathogenesis [3,4]. 

EV-A71 belongs to the species *Enterovirus A* of the genus *Enterovirus*, in the family *Picornaviridae*. The viral genome is positive-sense, single-stranded RNA with approximately 7400 base pairs in length which is encapsidated within an icosahedral protein shell. The genome organization is unique with untranslated regions at 5′ and 3′ termini, designated as 5′ UTR and 3′ UTR, respectively, which flank a single large open reading frame (ORF) encoding viral polyprotein [5]. Importantly, the 5′ UTR comprises a tertiary RNA structure, namely, the internal ribosomal entry site (IRES). IRES is responsible for the interaction and recruitment of cellular IRES trans-activating factors (ITAFs) and ribosomal complex to initiate the viral genome translation in a cap-independent manner [5]. However, the defined structure and functional domains of the EV-A71 IRES have not been resolved. The information on the structure and the functional domains can offer knowledge on the viral replication mechanism and the involved cellular factors, which would be targeted for further studies and the development of the effective and specific therapeutic measures. 

IRESs of picornaviruses have been classified into five types (types I to V) according to the differences in their conserved motifs and domains as well as the types of the host proteins that interact with them [6,7]. The enterovirus genome carries type I IRES which is highly conserved among the members of the genus [8,9]. Although the essential roles in the genome translation of enterovirus IRES have been studied, its detailed mechanism has not yet been elucidated. Resolving the crystal structure of a molecule of interest can provide information on its biological organization, function, and mechanism [10]. To gain a higher quality of the crystal, the co-crystallization of the target molecule bound with a specific antibody has been proven to assist the crystallography, the so-called, chaperone-assisted crystallography [11]. Most of the researchers studied the molecular details of the interactions between antibodies and EV-A71 proteins [12,13]. Therefore, there is very little information about the interaction of antibodies with RNA. The generation of antibodies against RNA by traditional technologies such as hybridoma technology is challenging because of the intrinsic instability and less immunogenicity of the RNA [14]. However, the advent of antibody phage display technology has made the in vitro isolation and production of anti-RNA antibodies become more achievable. In this study, we demonstrated the experimental approaches for isolating mouse single-chain variable fragment (scFv) antibodies specifically bound to EV-A71 IRES and characterizing their interaction. The obtained scFv antibody warrants its further development for use as a potential biological tool in the study of the structural biology of the EV-A71 RNA genome. Moreover, the gained knowledge will provide a better understanding of the mechanism of EV-A71 genome translation and those of other enteroviruses with implications for the development of specific interventions and treatments for enteroviral infections. The details of the methodology and results are mentioned herein. 

## 2. Results

### 2.1. Domains IV, V, and VI Were Structurally Conserved Regions of EV-A71 IRES

**Figure 1:**
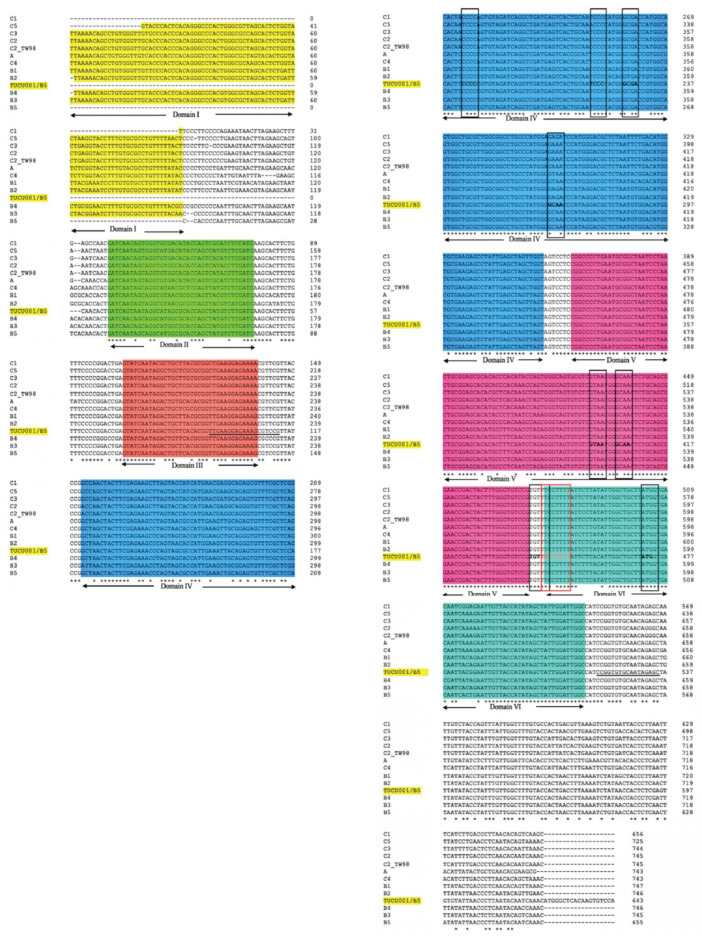
Multiple alignments of 5′ UTR of the major 12 genotypes of EV-A71 and TUCU001/B5 are performed by Clustal Omega. The identical nucleotides are indicated by the asterisks at the bottom of the alignments, and gaps (-) are introduced to optimize the alignments. Domains II to VI of enteroviral type I IRES on the multi-aligned 5′ UTR sequences are annotated by comparing them with C2_TW98 template sequences. The nucleotides underlined in domain III and downstream of domain VI were primer annealing sites for amplification of the interested domains IV, V, and VI. Conserved motifs are indicated by a black and red colored box. Areas colored in yellow, green, orange, blue, pink, and teal indicated 5′ UTR domains I to VI, respectively. * indicated identical nucleotides. - indicated gap of unaligned nucleotides.

**Figure 2:**
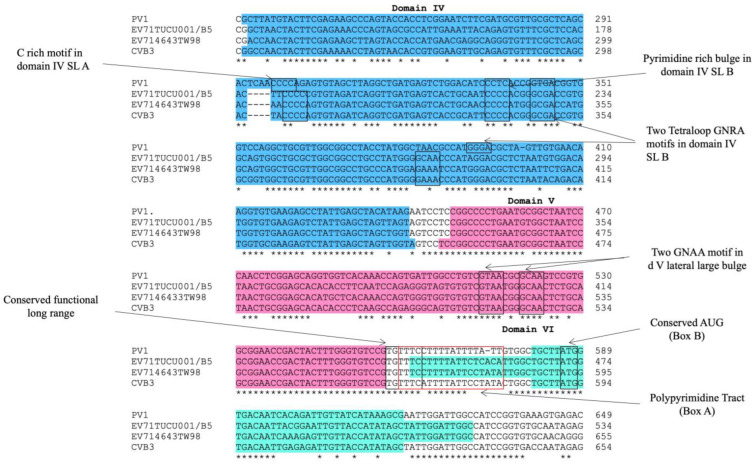
Multiple alignments of IRES domains IV, V, and VI of EV-A71 strain TUCU001/B5, EV-A71 strain 4643TW98 (or C2_TW98), human poliovirus (PV1), and coxsackievirus B (CVB3) to annotate signature motifs of type I IRES. Areas colored in blue, pink, and teal indicated IRES domains IV to VI, respectively. * indicated identical nucleotides. - indicated gap of unaligned nucleotides.

**Figure 3:**
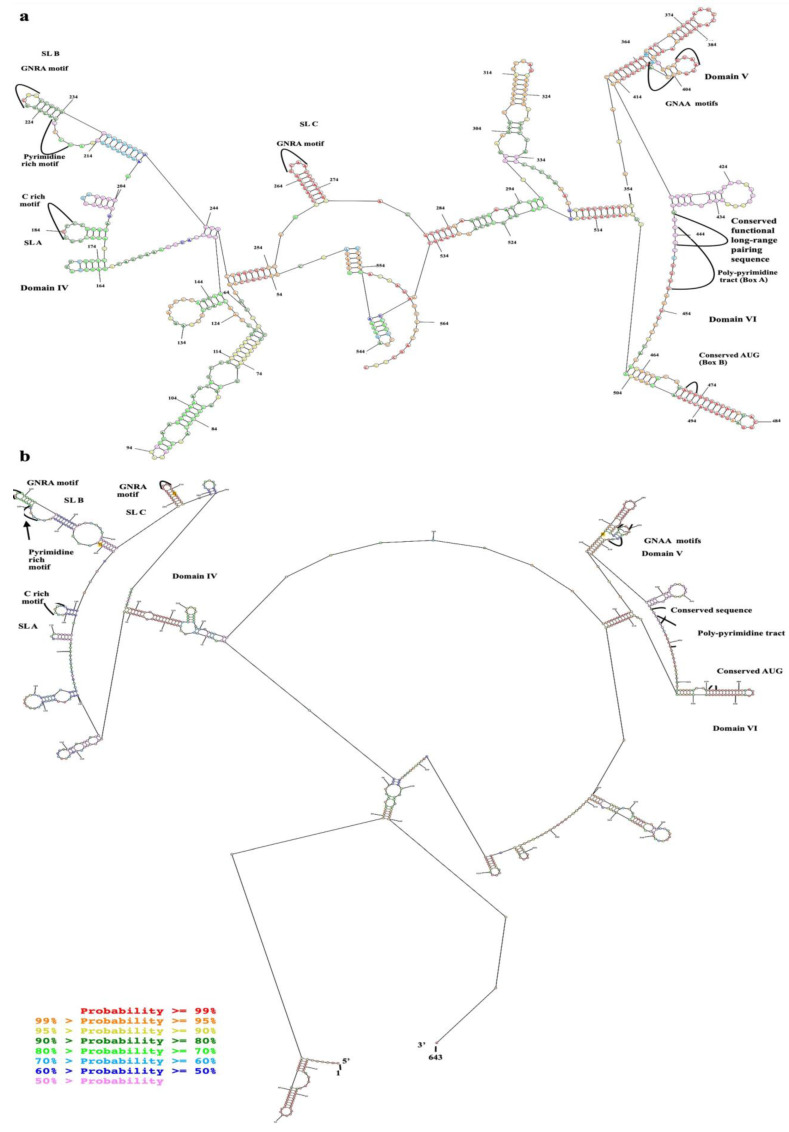
Predicted secondary structure of EV-A71 TUCU001/B5 IRES; (**a**) domains IV, V, and VI with flanking nucleotides from the plasmid which were in vitro transcribed together with the target sequence (total 528 nucleotides) and (**b**) the full-length IRES. Nucleotide numberings were annotated according to the 5′UTR sequence multiple alignments. The colors of each dot represented the % probability of the base-paring as indicated in the (**b**).

The alignment of the 5′ UTR sequences of EV-A71 TUCU001/B5 and a representative strain from each genotype of EV-A71 with the well-annotated sequence from EV-A71 C2_TW98 indicated that IRES, encompassing domains II to VI, were conserved among EV-A71, as they shared 85.48% sequence identity (Figure 1). From the domain annotation, it was found that the 5′ UTR of the EV-A71 strain TUCU001/B5 had complete domains II to VI which encompassed the whole sequence of IRES but lacked the complete domain I. The domains IV, V, and VI had the eligible sequence similarity. Therefore, the IRES domains IV, V, and VI were chosen for further analysis. The signature motif sequences of the domains IV, V, and VI of type I IRES were annotated by comparing them with the sequences from PV1, CVB3, and EV-A71 C2_TW98. It was found that IRES domains IV, V, and VI were located at the nucleotide positions 120 to 324, 332 to 440, and 445 to 515, respectively, on the EV-A17 TUCU001/B5 5′ UTR (Figure 2). The signature motifs conserved in the enteroviral type I IRES were identified in the IRES domains IV, V, and VI of the EV-A71 strain TUCU001/B5. For IRES domain IV, three conserved motifs, namely, the C-rich motif, pyrimidine-rich bulge, and tetraloop GNRA motif were identified. One C-rich motif and one pyrimidine-rich bulge were located at nucleotide positions 183 to 186 (_183_CCCC_186_) and 217 to 221 (_217_UCCCC_221_), respectively. There were two tetraloop GNRA motifs identified at nucleotide positions 226 to 229 (_226_GCGA_229_) and 266 to 269 (_266_GCAA_269_). In domain V, two conserved GNAA motifs were annotated at nucleotide positions 397 to 400 and 404 to 407. In domain VI, three conserved motifs including the functional long-range pairing sequence, pyrimidine tract/Box A/Yn, and silent AUG (Box B) were identified at nucleotide positions 441 to 445, 443 to 451, and 471 to 473, respectively. Taken together, the IRES domains IV, V, and VI of the EV-A71 were suitable for use as the target RNA antigen to produce the specific scFv monoclonal antibodies. To validate whether only the three domains could form secondary RNA structure, the predicted secondary structures of the selected IRES domains IV to VI of EV-A71 TUCU001/B5, together with flanking nucleotide regions on the cloning plasmid, which were to be in vitro transcribed, were compared with the whole IRES (the domain II to VI of 5′ UTR) (Figure 3a). It was found that the domains IV to VI with the flanking regions could form the secondary RNA structures of domains V and VI similar to the respective domains predicted on the full-length IRES (Figure 3b). While domain IV showed low similarity but had all the domain signature motifs (Table 1), taken together, it could be concluded that the IRES domains IV to VI of EV-A71 were structurally conserved among EV-A71 strains and were appropriate for use as the bio-panning antigens in the phage selection in terms of structural integrity. 

### 2.2. Production of EV-A71 IRES Domains IV, V, and VI RNA by In Vitro Transcription and Biotinylation 

**Figure 4:**
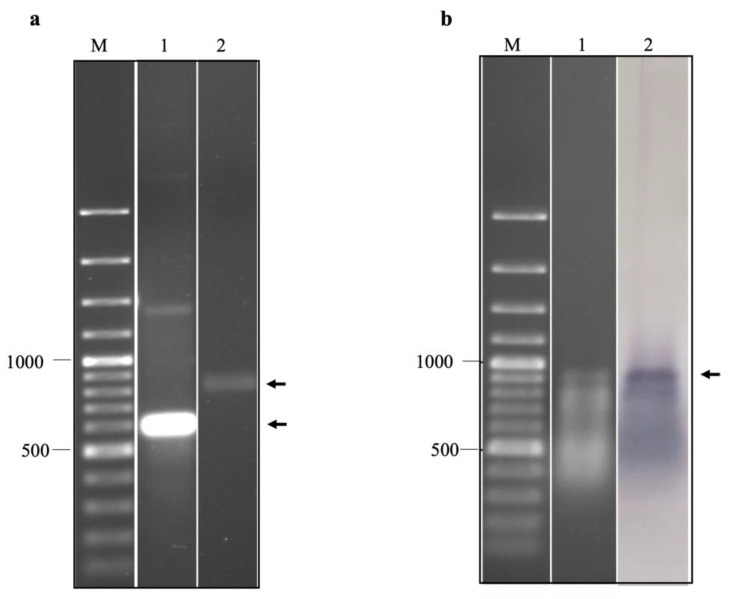
(**a**) Agarose gel electrophoresis of in vitro transcripts of EV-A71 TUCU001/B5 IRES domains IV, V, and VI prepared under non-denaturation (lane 1) and relaxed conditions (lane 2). (**b**) agarose gel electrophoresis (lane 1) and northern blot analysis (lane 2) of biotinylated in vitro transcripts of EV-A71 TUCU001/B5 IRES domains IV, V, and VI prepared under relaxed conditions. Lanes M; 100 bp plus DNA ladder. The expected bands of the target products are indicated by arrows.

The purified in vitro transcripts of EV-A71 IRES domains IV, V, and VI were prepared in non-denaturation and relaxed conditions, which were then resolved by non-denaturation agarose gel electrophoresis (Figure 4a). It was found that the in vitro transcripts prepared under the non-denaturation condition migrated in the agarose gel faster than those of the formamide/formaldehyde relaxed sample. It could be implied that the in vitro RNA transcripts of EV-A71 IRES domains IV, V, and VI had secondary structures. By northern blotting assay, it was found that the biotinylated in vitro transcripts were intact and successfully labeled with biotin (Figure 4b). Taken together, the biotinylated in vitro transcripts of EV-A71 IRES domains IV, V, and VI were suitable for biopanning.

### 2.3. Characteristics of the Monoclonal scFv Antibody Specifically Binds EV-A71 IRES 

**Figure 5:**
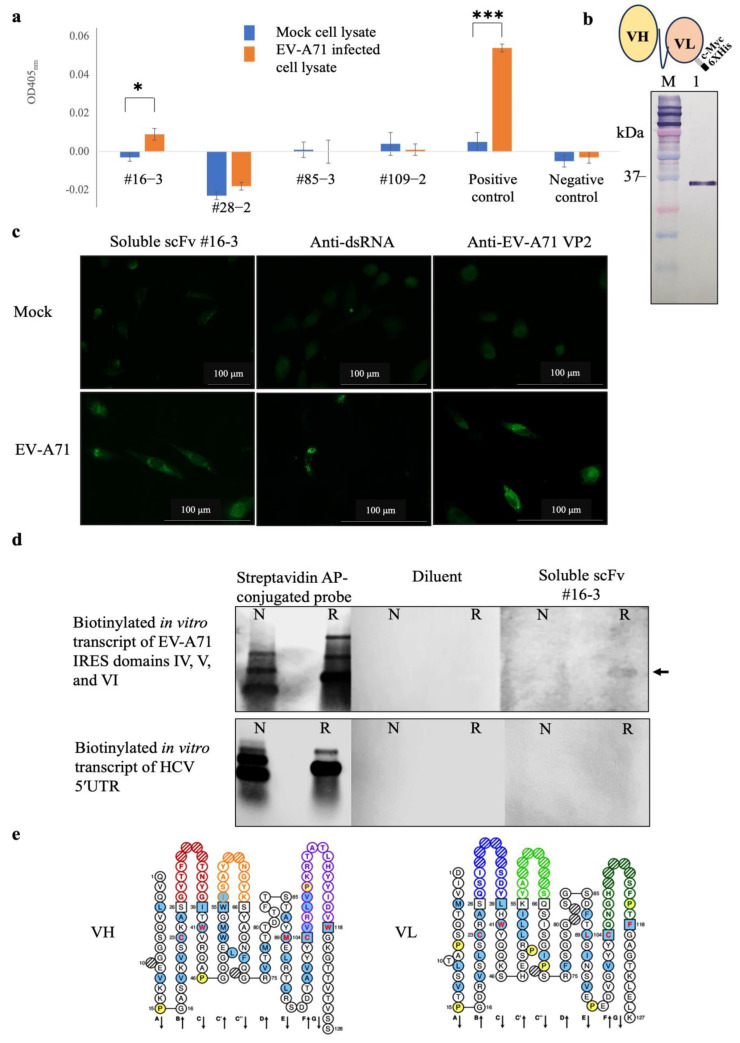
Results of (**a**) screening of scFv-phages that bound to EV-A71 infected cell lysate by indirect ELISA of representative clones, (**b**) Western blot analysis of the purified soluble scFv #16-3 by mouse monoclonal anti-C Myc antibody and schematic drawing of scFv molecule fused with cMyc and 6× His epitope tags, (**c**) binding activity of soluble scFv #16-3 to RD cell monolayer infected with EV-A71 tested by immunofluorescence assay, (**d**) binding activity of soluble scFv #16-3 to biotinylated in vitro transcripts of EV-A71 IRES domains IV, V, and VI prepared under non-denaturation and relaxed conditions (as indicated by N and R, respectively) tested by northern blot analysis, and (**e**) amino acid sequences of the predicted VH and VL domains of the scFv #16-3; the colored loops represent CDRs [15]. In indirect ELISA, the cell lysate probed with anti-dsRNA antibody and M13K07 wild-type phage served as a positive and negative control, respectively. In the immunofluorescence assay, RD cell monolayer infected with EV-A71 incubated with anti-dsRNA and anti-EV-A71 VP2 antibodies served as positive controls. Normal RD cells probed with soluble scFv #16-3, anti-dsRNA, and anti-EV-A71 VP2 antibodies served as mock controls. In northern blot analysis, the blotted antigens incubated with streptavidin AP-conjugated probe served as antigen control. The blotted membranes probed with diluent were negative control. Biotinylated in vitro transcript of 5′ UTR HCV served as an irrelevant antigen. The data of (**a**) were derived from three independent experiments and expressed in mean ± SD and analyzed by *t*-test method. Levels of statistically significant difference at *p*-value  <  0.05 and *p*-value  < 0.001 were indicated by * and ***, respectively.

scFv-phages that might potentially bind to irrelevant antigens were subtracted by sequentially incubating the scFv antibody library with purified biotin, biotinylated HCV 5′ UTR, and biotinylated EV-A71 2B transcripts immobilized on the magnetic beads. After magnetic bead separation, the unbound scFv-phages were incubated with the biotinylated in vitro transcript of EV-A71 IRES domains IV, V, and VI immobilized on the magnetic beads. The target antigen-bound scFv-phages were collected by magnetic bead separation and allowed them to transduce *E. coli* with phagemids inserted with the respective *scfv* coding sequences. The positively selected scFv-phages were rescued and propagated from the individually transformed *E. coli* colonies. It was found that the scFv-phages could be rescued from the individual 121 clones of the transformed *E. coli*. All 121 clones of scFv-phage were screened for binding activity to native viral antigens with EV-A71 infected cell lysate by indirect ELISA. It was found that only one scFv-phage clone number #16-3 (scFv-phage #16-3) showed a significant binding activity to the EV-A71 infected cell lysate, which was comparable to the positive control, anti-dsRNA antibody, but not to the mock RD cell lysate (Figure 5a). While the M13KO17 wild-type phages showed negligible signals to the EV-A71, infected cell lysate served as a negative control. It suggested that the scFv-phage #16-3 might bind native EV-A71 antigens. 

To confirm the binding activity, the scFv-phage #16-3 was reformatted from antibody-displayed phage to a free-molecule of soluble scFv (Figure 5b). The purified soluble scFv #16-3 was verified for the binding activity with the native EV-A71 antigen in RD cells infected with EV-A71 by immunofluorescence assay. It was found that the soluble scFv #16-3 bound to the antigen in the EV-A71 infected cells but did not bind to the antigen in the mock cells. The EV-A71 VP2 proteins and the EV-A71 replicative dsRNA were detected only in the infected cells (Figure 5c). It was confirmed that the soluble scFv #16-3 was bound to native EV-A71 antigen in the infected-RD cells. Then, the soluble scFv #16-3 was tested for binding to the authentic antigen, biotinylated in vitro transcript of EV-A71 IRES domains IV, V, and VI, by northern blot analysis. It was shown that the soluble scFv #16-3 bound only to the relaxed biotinylated EV-A71 IRES domains IV, V, and VI transcripts and not to the non-denatured antigen and both forms of the biotinylated HCV 5′UTR transcripts (Figure 5d). 

Moreover, the scFv #16-3 was predicted to be a complete antigen-binding domain as its nucleotide sequence contained a variable domain of the heavy-chain (*vh*) and the light-chain (*vl*) (Figure 5e). Taken together, it was confirmed that the scFv #16-3 was specifically bound to the EV-A71 IRES and did not bind to the non-type I IRES. 

### 2.4. Computational Prediction of Interaction between scFv and EV-A71 IRES

**Figure 6:**
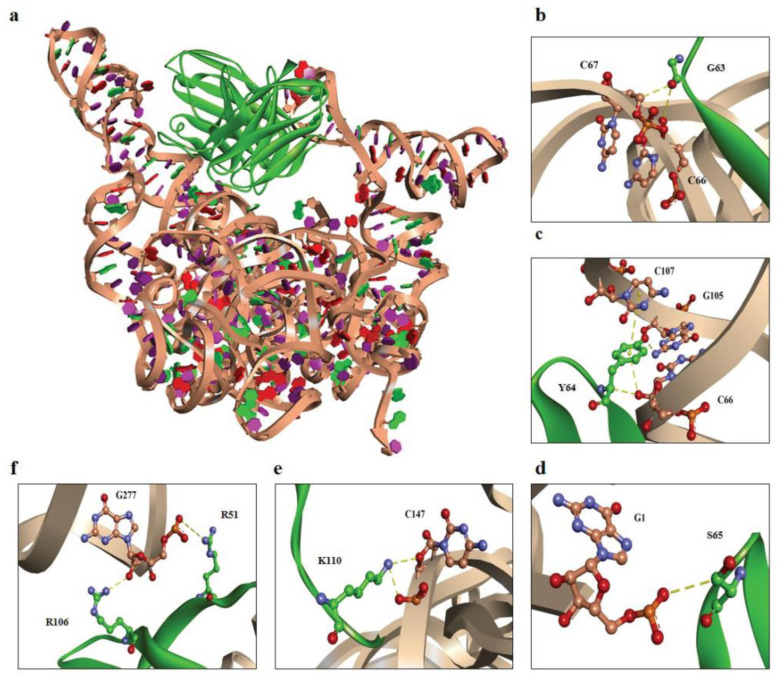
Computerized interactions between modeled EV-A71 IRES domains IV, V, and VI and scFv #16-3. (**a**) CDR loops and FRs of scFv #16-3 (green) interact with the EV-A71 IRES domains IV, V, and VI (beige). (**b**–**f**) representatives of the interactive residues between scFv #16-3 (green) and EV-A71 IRES domains IV, V, and VI (beige). (**b**) G63 and (**c**) Y64 are residues on VH-CDR2, (**d**) S65 residue is on VL-CDR2, (**e**) K110 is on VH-CDR3, and (**f**) R106 and R51 are residues on VH-CDR3 and VL-FR2, respectively. Non-covalent bonds are indicated by yellow-colored dots.

The molecular docking approach predicted the interaction between scFv #16-3 and EV-A71 IRES domains IV, V, and VI. It was shown that both VH and VL of scFv #16-3 interacted with the domains IV and V by a variety of non-covalent bonds (Figure 6 and Table 2). Moreover, the preference bindings for serine (S), tyrosine (Y), glycine (G), lysine (K), and arginine (R) were observed in the regions of the scFv #16-3 CDRs interacting with the EV-A71 IRES.

## 3. Discussion

It has been proven that the structurally conserved motifs of type I IRES of enteroviruses and their integrity play a crucial role in the biological functions leading to a successful infection [16]. Interaction of the enteroviral IRES with the host proteins facilitates viral genome translation. The elucidation of the IRES-host interaction will provide a further understanding of the process of the genome translation of EV-A71 and other enteroviruses. Resolving the crystal structure of the EV-A71 IRES might provide information on its organization, functions, and mechanism of biological functions. To obtain the high-quality crystal, the target molecule bound with a specific antibody has been employed in the co-crystallization approach [17]. It has been proven that the antibodies can accelerate the precipitation of the protein crystals by acting as a chaperone called chaperone-assisted crystallography [18]. However, the production of the antibody against IRES is challenging because of the low immunogenicity and the less stability of the RNA molecule. To overcome these drawbacks, this study employed the in vitro selection and production of monoclonal antibodies by phage display technology using synthetic structurally conserved motifs of EV-A71 IRES as the target antigen. Analyses of the sequence homology and the structure prediction revealed that IRES type I was conserved among EV-A71 genotypes, especially in the domains IV, V, and VI. The secondary structures of the domains IV, V, and VI of EV-A71 IRES were predicted and compared with the respective domains in the whole IRES. The analyzed sequence was nucleotides of domains IV, V, and VI (395 nucleotides in length) together with flanking regions of the multi-cloning site in the recombinant plasmid to yield a total of 528 nucleotides in length. The secondary structures of the 528 bp-IRES and whole IRES sequences were predicted with the minimal free energy of −192.6 kcal/mol and −227.1 kcal/mol, respectively. It indicated the thermodynamic stability of the predicted RNA secondary structures. Thus, it could be implied that the domains IV, V, and VI of EV-A71 IRES inserted in the cloning vector downstream to the T7 promotor would successfully produce the transcripts carrying the intact secondary structure mimic to the natural viral RNA by in vitro transcription assay. It was envisaged that the synthetic domains IV, V, and VI might be the suitable target antigen for the isolation of antibodies specific to EV-A71 IRES.

In this study, we employed the naïve mouse single-chain variable fragment phage display (scFv-phage) library to in vitro select antibodies specific to the EV-A71 IRES domains IV, V, and VI. The in vitro selection of the antigen-specific antibodies from the phage display library, namely, biopanning, could bypass immune regulation that normally happens in vivo during the clonal selection of the epitope-specific B cells [19]. Hence, it could overcome the low immunogenicity of RNA. The library size utilized in this experiment was approximately 1.3 × 10^8^ with substantial antibody sequence diversity, which was comparable to the other non-immune libraries previously reported [20]. Therefore, the library has been reported for the successful selection of scFv specific to a variety of antigens including EV-A71 virion protein 2 (VP2) and Di^a^ blood group antigen [20,21]. Similarly, a related study using a human naïve antigen-binding fragment (Fab) antibody combinatorial library which had a size of approximately 5.0 × 10^8^ could be used to successfully isolate anti-RNA antibodies [22]. Therefore, the naïve scFv-phage library used in this study which has a magnitude of diversity comparable to those synthetic libraries might have a chance to select antibodies specific to EV-A71 IRES RNA antigen. However, it was found that only one clone, scFv-phage #16-3, gave a low but significant ELISA signal to the EV-A71 antigen in the infected RD cell lysate. This was 0.83% of the total tested scFv-phage clones (121 clones). A possible reason for the low positive rate of the antigen binder might be due to the low frequencies of the RNA-specific scFv repertoire contained in the library. The low ELISA signals from the viral antigen control, which was the infected-cell lysate-coated wells incubated with anti-dsRNA antibody, indicated fewer proportions of dsRNA contained in the lysate. Alternatively, the potential incomplete inactivation of RNAse by RNAse inhibitors might cause the degradation of the RNA. Nevertheless, it indicated that the selected scFv-phage #16-3 had the potential to bind to the viral RNA. This speculation was verified by northern blot analysis using soluble scFv #16-3 to detect biotinylated EV-A71 IRES domains IV, V, and VI. It was found that scFv #16-3 bound specifically only to the relaxed biotinylated in vitro transcripts of EV-A71 IRES domains IV, V, and VI and not to the non-denatured form. It was hypothesized that the relaxed IRES could be renatured upon non-denaturing agarose gel electrophoresis and exposed epitope bound by the scFv #16-3 as detected by northern blotting. While the non-denatured RNA might be compactly folded and masked for the epitope bound by scFv #16.3 resulting in a negligible band in northern blotting. In the biopanning step, the folded state of the in vitro transcribed IRES in the solution containing Mg^2+^ might be different from those used as antigen in the northern blot analysis. It has been ported that the folded RNA in the solution containing Mg^2+^ showed a flexible and solvent-accessible surface area in its native state [23]. Hence, we speculated that the IRES antigen used in biopanning could be folded in the solution and exposed epitopes for selecting the scFv-phages. 

The dynamic of the viral RNA conformation throughout viral replication has been reported [24,25]. Within the replication organelles in the cytoplasm of the virus-infected cells, the epitope of the EV-A71 viral IRES might be exposed at a particular phase of the viral replication process such as RNA circularization [26]. This type of RNA conformational reorganization might facilitate the binding interaction of the scFv #16-3 to the EV-A71 IRES domains. This notion warrants further experimental validation. By immunofluorescence assay, the soluble scFv #16-3 could detect the EV-A71 infection, comparable to the anti-dsRNA antibody capable of probing the replicative form of many positive-stranded RNA viruses [27,28]. It has been reported that the paratope of anti-RNA antibodies is formed by a shape-complementary adaptation of CDRs especially CDR-H1 to H3 and CDR-L3 to a short stretch of nucleotides such as helix and bulge or even by direct non-covalent interactions with the base and sugar ring but not a huge RNA secondary structure [29,30]. The molecular docking predicted interaction between scFv #16-3 and EV-A71 IRES domains IV and V was facilitated by both VH and VL through non-covalent bonds. The preferences for the IRES-interacting residuals including serine (S), tyrosine (Y), glycine (G), lysine (K), and arginine (R) were observed on the CDRs of VH and VL of the scFv #16-3. Previous studies demonstrated that the Fab selected from the synthetic phage libraries preferentially employed these amino acid residues to create contact sites with an epitope on the target RNA antigen [31,32]. The synthetic Fab phage display libraries that had codon bias for S, Y, G, K, and R residues at CDRs have been achieved to select the antibody specific to type III IRES in hepatitis A virus (HAV-like IRES) and type IV IRES in HCV (HCV-like IRES) [17,33]. However, the predicted interaction between the scFv #16-3 and EV-A71 IRES warrants further experimental validations such as mutagenesis and crystallography. It could be concluded that the scFv antibody specific to the structurally conserved domains of EV-A71 type I IRES was successfully produced. The obtained scFv antibody has the potential for further development to be used as a biological tool for studying the structural biology of the EV-A71 RNA genome and the diagnostic reagent of EV-A71 infection. These notions warrant experimental verification with at least other enteroviruses such as poliovirus and coxsackieviruses.

## 4. Materials and Methods

### 4.1. Virus

Human rhabdomyosarcoma (RD) cells (JCRB9072) were cultured in Dulbecco’s modified Eagle’s medium (DMEM, Biochrom, Cambridge, UK) supplemented with 10% fetal bovine serum, L-glutamine, and penicillin/streptomycin antibiotics (Gibco, Billings, MT, USA). The cells were incubated at 37 °C in humidified air containing 5% CO_2_. Enterovirus A71 (EV-A71) subgenotype B5 kept in our laboratory, namely TUCU001/B5, was propagated in RD cells and its titers were determined by median cell culture infectious dose (CCID_50_) as described previously [20].

### 4.2. Selection and Preparation of EV-A71 IRES Antigen

#### 4.2.1. Identification of Conserved Regions of EV-A71 IRES

Complementary DNA (cDNA) sequences of the 5′ UTR of 11 representative strains from each major EV-A71 genotypes, A, B1-B5, and C1-C5, and one prototype sequence of EV-A71 genotype C2, isolate 4643-TW98; designated C2_TW98, were retrieved from NCBI GenBank^®^ database (Table 3). In addition, 5′ UTR sequence of EV-A71 strain TUCU001/B5 was identified for the conserved domains of enteroviral type I IRES by multiply aligned with the selected 12 sequences using Clustal Omega (https://www.ebi.ac.uk/Tools/msa/clustalo/, accessed on 1 March 2022) of which C2_TW98 served as the template. The signature motifs of the individual domains of type I IRES of the EV-A71 strain TUCU001/B5 were identified by comparing with the well-annotated cDNA sequences of enteroviral type I IRES including reference sequences of human poliovirus (PV1; serotype 1 strain Mahoney, Accession no: NC_002058.3) and coxsackievirus B (CVB3; serotype B3 strain 28, Accession no: AY752944.2). The conserved regions of EV-A71 IRES were selected for use as antigen in the biopanning step. 

To ensure the correct folding of the selected domains of EV-A71 IRES upon in vitro production and subsequent experiments, the RNA secondary structures of the selected regions of EV-A71 IRES were predicted by web-based tools namely RNAstructure [34]. Based on thermodynamic folding, a structure with the lowest predicted free energy was refined by the free energy minimization for their folding at the default parameters including temperature at 37 °C, 1M NaCl, no divalent ion, and 5% suboptimality.

#### 4.2.2. Preparation of the Selected Domains of EV-A71 IRES for Biopanning 

The conserved regions of EV-A71 IRES were amplified from cDNA of the full-length genome of EV-A71 strain TUCU001/B5 inserted in pJET 1.2/blunt cloning vector, CloneJET PCR cloning kit (Thermo Scientific, Waltham, MA, USA), by high fidelity Profi-Taq DNA polymerase (Bioneer, Daejeon, Republic of Korea) using IRES-d IV forward primer (5′-TTG AAG GAG AAA GCG TCC G-3′) and IRES-d VI reverse primer (5′-GCT CTA TTG CAC ACC GG-3′). The purified PCR amplicons were ligated into pJET 1.2/blunt cloning vector (Thermo Scientific, CloneJET PCR cloning kit) according to the manufacturer’s instructions. After selection and verification, the recombinant plasmid was used as a template for synthesizing the conserved regions of EV-A71 IRES by T7 in vitro transcription assay using Ribomax™ Large Scale RNA production kit (Promega, Madison, WI, USA) according to the manufacturer’s instructions. Controls were in vitro RNA transcripts synthesized from recombinant plasmids inserted with 5′UTR of hepatitis C virus (kindly given by Dr. Monrat Chunlanetra, Faculty of Medicine Siriraj Hospital, Mahidol University, Thailand), and 2B coding sequence of EV-A71 kept in our laboratory. The in vitro RNA transcripts of 5′UTR of HCV containing type IV IRES and a coding sequence of EV-A71 2B served as non-type I IRES and irrelevant RNA controls, respectively. The purified in vitro RNA transcripts were conjugated with biotin at 3′ end using an RNA labeling kit (Pierce 3′ end biotinylation, Thermo Scientific, Waltham, MA, USA) according to the manufacturer’s instructions. The integrity of the biotinylated in vitro RNA transcripts was verified by non-denaturation agarose gel electrophoresis and northern blot analysis using streptavidin-AP conjugate. The RNA samples were mixed with RNA sample buffer containing formamide/formaldehyde and prepared under either non-denaturation or relaxed conditions and resolved by non-denaturation agarose gel electrophoresis. For the relaxed condition, the RNA transcript samples were mixed with RNA sample buffer at the ratio of 2:1 volume/volume and heated at 60 °C for 5 min then immediately placed on ice for 3–5 min.

### 4.3. Biopanning of Phage Display Single-Chain Variable Fragment Antibody (scFv)

#### 4.3.1. Preparation of Antibody Phage Display Library

The naïve phage display library containing a repertoire of mouse single-chain variable fragment antibodies (scFv-phages) which had been constructed and described previously by [20] was used to select the IRES-specific antibody. The library had approximately 1.3 × 10^8^ antibody diversity and ~1.4 × 10^12^ colony-forming units (CFU)/mL of scFv-phages [20]. To retain the integrity of the IRES during biopanning, the scFv-phage library was prepared in in vitro RNA assembly buffer (1.7 mM Mg [CH_3_ COO]_2_, 100.0 mM CH_3_CO_2_K, and 25.0 mM HEPES) supplemented with RNAse inhibitor (Ribolock, Thermo Scientific, Waltham, MA, USA). The library was mixed in 5.8% PEG-8000/NaCl and kept at 4 °C for 16 h. The scFv-phages were precipitated by centrifugation at 13,000× *g*, 4 °C for 15 min. The precipitates were washed with the in vitro RNA assembly buffer by centrifugation and then resuspended with the same buffer.

#### 4.3.2. Immobilization of Antigens

For biotin antigen, 10 μg of biotin (BAC-SulfoNHS, Sigma, Steinheim, Germany) was mixed with carbonate-bicarbonate coating buffer pH 9.6 in a final volume of 100 μL and coated in a well of ELISA plate and incubated at 37 °C overnight. The wells were then washed three times with Tris-buffered saline containing 0.05% Tween-20 (0.05% TBS-T) and blocked with 3% BSA in 0.05% TBS-T at 37 °C for 1 h in a moisture chamber. 

For RNA antigens, biotinylated RNA antigens including the RNA transcripts of EV-A71 IRES target antigen, HCV 5′ UTR, and EV-A71 2B coding sequence were coated on streptavidin magnetic beads (Pierce^TM^ Streptavidin magnetic beads, Thermo Scientific, Waltham, MA, USA). Firstly, 20 μg of the streptavidin magnetic beads were washed with 0.05% TBS-T and collected by the DynaMag Spin (Life Technologies, Waltham, MA, USA). The empty surface areas on the beads were blocked with 3% BSA followed by equilibration with in vitro RNA assembly buffer on a rotator for 15 min. The beads were collected and separately incubated with 2 μg of biotinylated EV-A71 IRES (11.78 pmol), biotinylated HCV 5′ UTR (14.3 pmol), biotinylated EV-A71 2B transcripts (15.78 pmol), which were prepared in the in vitro RNA assembly buffer for 1 h at ambient temperature with rotating. Thereafter, the beads were washed three times with TBS-T (0.05%) to eliminate the unbound antigens. The RNA-bound magnetic beads were resuspended in the in vitro RNA assembly buffer.

#### 4.3.3. Biopanning

The so-prepared library containing 2.3 × 10^6^ CFU scFv-phages was incubated in the biotin-coated well at 37 °C for 30 min. Then, the supernatant was collected and consecutively performed negative biopanning with the other RNA controls, including biotinylated HCV 5′ UTR and biotinylated EV-A71 2B transcripts immobilized on the magnetic beads for 1 h with rotation. Finally, the subtracted scFv-phage library was subjected to positive biopanning. The unbound scFv-phages were then incubated with IRES-bound magnetic beads. After incubation, the unbound phages were washed with 0.05% TBS-T and eliminated by magnetic separation. The bound phages were eluted from the beads by treatment with trypsin [20]. The eluate was incubated with log-phase XL-1 Blue *E. coli* at 37 °C for 15 min to facilitate transduction of the phagemid containing *scfv* coding sequences into the bacterial cells. Bacterial transformants harboring *scfv*-inserted phagemids were selected by growing on the selective agar plate and screening by direct colony PCR. The scFv-phages were rescued from the individual transformed *E. coli* culture by co-infecting the bacteria with M13KO7 helper phages (GE Healthcare Life Sciences, Chicago, IL, USA). The titers of the rescued phages were determined and normalized by colony-forming count. 

### 4.4. Binding of the Selected scFv-Phages to EV-A71-Infected Cell Lysate

Rhabdomyosarcoma (RD) cell monolayer was infected with EV-A71 isolate TUCU001/B5 at MOI of 5. At 12 h post-infection, the culture medium was aspirated, and the infected cell monolayer was lysed by adding 1 mL of pre-chilled RIPA lysis buffer (150 mM NaCl, 5 mM EDTA, 25 mM Tris-HCl pH 7.6, 1% Triton X-100, 1% sodium deoxycholate, 0.1% SDS) supplemented with RNase inhibitor and incubated on ice for 30 min. After centrifugation, the infected cell lysate was collected. Cell lysate prepared from the uninfected cells served as mock control. The infection of the so-prepared cell lysate was verified by real-time PCR. The cell lysates were mixed with PBS coating buffer and immobilized in wells of Nunc Immobilizer Amino Strips (Thermo Scientific, Waltham, MA, USA) at ambient temperature for 1 h on a gentle agitator followed by overnight incubation at 4 °C. Thereafter, the wells were washed 3 times with 0.05% PBS-T prepared in DEPC-treated water and incubated with 1% hydrogen peroxide in DEPC-treated water for 15 min for the inactivation of the endogenous peroxidase. After that, the wells were incubated with 1× nucleic acid blocking solution (Roche, Basel, Switzerland) for 1 h followed by 5% FBS in PBS for another 1 h. Individual scFv-phage clones were normalized to 10^8^ CFU through dilution with PBS-T. One hundred microliters of the diluted scFv-phages were incubated with the cell lysate-coated wells (both infected and uninfected) at 37 °C for 1 h and then washed 5 times with PBS containing 0.5% Tween 20. 

The ELISA signals were revealed by incubating the contents in the wells with mouse anti-M13 antibody (GE Healthcare, Chicago, IL, USA) and goat anti-mouse HRP conjugated antibody (KPL, Gaithersburg, MD, USA) followed by adding ABTS substrate. The colorimetric signals were measured at an optical density of 405 nm (OD405 nm) by a plate reader (Varioskan Flash, Thermo Scientific, Waltham, MA, USA). The wells incubated with Luria-Bertani broth and M13KO7 wild-type phage served as background and negative control, respectively. Moreover, the viral antigen control (also known as positive control) was infected-cell lysate-coated wells incubated with mouse anti-double-stranded RNA (dsRNA) monoclonal antibody (J2 clone from English and Scientific Consulting Kft, Szirák, Hungary) followed by goat anti-mouse HRP conjugated secondary antibody (KPL, Gaithersburg, MD, USA). The scFv-phages that yielded OD405nm values with infected cell lysate greater than 2 times over the mock cell lysate were selected. 

### 4.5. Production and Characterization of Soluble scFv Antibody

The *scfv* coding sequence of the selected clone was subcloned from the pSEX81 phagemid into the pOPE101 plasmid (Progen Biotechnik GmbH, Heidelberg, Germany) as described previously [20]. The insertion of the *scfv* coding sequence was verified by DNA sequencing. The complementarity determining regions (CDRs) and immunoglobulin framework regions (FRs) of the *scfv* sequence was predicted using the IMGT/V-QUEST tool (http://www.imgt.org/, accessed on 1 March 2022) [35]. The transformed XL-1 Blue *E. coli* harboring recombinant pOPE101 inserted with *scfv* coding sequence was grown under isopropyl β-D-1-thiogalactopyranoside (IPTG) induction. The soluble scFv was purified from *E. coli* lysate using Ni–NTA affinity chromatography. The purified soluble scFv was checked for integrity by SDS-PAGE and Western blot analysis using mouse monoclonal anti-c-Myc (BioLegend, San Diego, CA, USA).

### 4.6. Antigenic Specificity of the Soluble scFv Antibody 

The soluble scFv was tested for binding to the native viral antigen in EV-A71-infected RD cells and biotinylated in vitro RNA transcript of EV-A71 IRES by immunofluorescence assay and northern blot analysis, respectively. For immunofluorescence assay, EV-A71 infected RD cell monolayer was incubated with soluble scFv for 1 h followed by mouse anti-C-Myc and Alexa Flur 488 conjugated goat anti-mouse antibody. The mock control was an uninfected monolayer cell incubated with soluble scFv. The positive control was an infected-monolayer cell probed with a mouse anti-dsRNA monoclonal antibody. The infection control was infected-monolayer cell probed with an anti-EV-A71 monoclonal antibody specific to VP2 protein (Clone 422-8D-4C-4D 10F0 from Bio-Rad, Hercules, CA, USA). For northern blot analysis, the biotinylated in vitro transcripts of EV-A71 IRES and HCV 5′ UTR were normalized at 0.8 μg and prepared under both non-denaturation and relaxed conditions. To relax the secondary structure of RNA transcripts, an equal volume of RNA sample and sample buffer (1.3% formaldehyde and 0.6× MOPS buffer) were mixed and heated at 60 °C for 5 min and then immediately chilled on ice for 5 min. The RNA samples were resolved in 1.2% agarose gel by electrophoresis using 1× MOPS buffer. The biotinylated RNA samples and controls were transferred by traditional upward, passive capillary blotting into a positively charged nylon membrane (Amersham Hybond-N+). UV crosslink at 245 nm bulb with 120 mJ/cm^2^ for 1 min was carried out immediately to immobilize the RNA on the membrane. The blotted membrane was blocked with 1× blocking buffer (Roche, Basel, Switzerland). The blotted membranes were incubated with approximately 10 μg of the soluble scFv followed by mouse anti-C-Myc antibody and AP-conjugated goat anti-mouse antibody. Then, the immune complex band was visualized by BCIP/NBT substrate. The blotted membranes incubated with streptavidin-AP probe and diluent served as positive and negative control, respectively. 

### 4.7. Homology Modeling and Intermolecular Docking 

The protein structure and function of the selected scFv were predicted by molecular modeling of its DNA sequence using the I-TASSER server [36,37,38]. Then, the model was transformed to be more relevant to the native state by ModRefiner server [39]. The interface contacts between EV-A71 IRES and its cognate scFv were predicted by HDOCK server [40,41,42,43,44]. The folded structure of the domains IV to VI EV-A71 IRES was also modelled and selected by HDOCK. The significant docking clusters of the interactive residues with the lowest local energy were selected. The interactive amino acid residues were visualized by BIOVIA, Dassault Systèmes, Discovery Studio Visualizer version 19.1.0.18287, San Diego: Dassault Systèmes, release 2019 (http://www.3ds.com/support/download/, accessed on 1 March 2022). 

### 4.8. Nucleotide Sequence

The nucleotide sequence of scFv reported in this paper has been submitted to the NCBI GenBank^®^ database with accession number OK642583 (https://www.ncbi.nlm.nih.gov/nuccore/OK642583, accessed on 1 March 2022). 

## Figures and Tables

**Table 1:** Comparison between signature motifs on the predicted RNA secondary structures of the selected domains IV to VI and the whole IRES sequence.

Motifs	Domains IV to VI with Flanking Regions of Cloning Plasmid (528 nts)	Whole IRES (Domains II to VI) (643 nts)
Minimal free energy (kcal/mol)	−192.6	−227.1
C rich motif (CCCC) of domain IV	in hairpin loop	in hairpin loop
Pyrimidine rich bulge (UCCCC) of domain IV	in lateral bulge	in lateral bulge
1st GNRA motif (GUCA) of domain IV	in hairpin loop	in hairpin loop
2nd GNRA motif (GGGA) of domain IV	in hairpin loop	in hairpin loop
1st GNAA motif (GUAA) of domain V	in hairpin loop	in hairpin loop
2nd GNAA motif (GCAA) of domain V	at the base of the hairpin	at the base of the hairpin
UGUUU in connecting region of domains V and VI	in multi-branch loop	in multi-branch loop
UUUCCUUUU (Box A) of domain VI	in multi-branch loop	in multi-branch loop
AUG (Box B) of domain VI	in stem/helix	in stem/helix

**Table 2:** Residues of the EV-A71 IRES bound by the residues and domains of scFv #16-3.

ScFv #16-3		EV-A71 IRES		Interactive Bond
Residue	Domain	Nucleotide	Domain	
Q1	VH-FR1	G277	V	Hydrogen Bond
V2	VH-FR1	U278	V	Hydrogen Bond
N62	VH-CDR2	C107	IV	Hydrogen Bond
G63	VH-CDR2	C66	IV	Hydrogen Bond
G63	VH-CDR2	C67	IV	Hydrogen Bond
Y64	VH-CDR2	C66	IV	π-Hydrogen Bond
Y64	VH-CDR2	G105	IV	Hydrogen Bond
Y64	VH-CDR2	C107	IV	π-π interaction
R95	VH-FR3	A95	IV	Salt Bridge, π-Alkyl
R106	VH-CDR3	G277	V	Electrostatic
K110	VH-CDR3	C147	IV	Electrostatic, Hydrogen Bond
R111	VH-CDR3	C56	IV	Electrostatic, Hydrogen Bond
R111	VH-CDR3	A57	IV	Electrostatic
R111	VH-CDR3	G145	IV	Electrostatic
R111	VH-CDR3	G146	IV	Electrostatic, π-Alkyl
T112	VH-CDR3	G108	IV	Hydrogen Bond
W118	VH-FR4	U278	V	Hydrogen Bond
S36	VL-CDR1	U54	IV	Hydrogen Bond
R51	VL-FR2	G277	V	Hydrogen Bond
S65	VL-CDR2	G1	IV	Hydrogen Bond
S69	VL-FR3	C276	V	Hydrogen Bond
R75	VL-FR3	G284	V	Electrostatic
E97	VL-FR3	G283	V	Hydrogen Bond
H108	VL-CDR3	A148	IV	π-Anion

**Table 3:** Sequences of the EV-A71 5′ UTR used in the multiple alignment analysis.

Genotype	Strain	GenBank^®^ Accession No.	Country
A	BrCr, prototype	U22521	USA
B1	Nagoya	AB482183.1	Japan
B2	MS/7423/87	U22522.1	USA
B3	SK-EV006	AB550334.1	Malaysia
B4	5666/sin/002209	AF352027.1	Singapore
B5	THA-EV71-019	JF738001.1	Thailand
C1	THA-EV71-002	JF738000.1	Thailand
C2	Hau1	KM077140.1	Taiwan
C2	4643-TW98	JN544418.1	Taiwan
C3	03-KOR-00	DQ341356.1	Republic of Korea
C4	SH12-036	KC570452.1	China
C5	2007-07364	EU527983.1	Taiwan

## Data Availability

The data presented in this study are available on request from the corresponding author. The accession numbers of 5′ UTR sequences of reference strains of human poliovirus (PV1), coxsackievirus B (CVB3), and EV-A71 used in the multiple alignment analysis were from the GenBank repository and mentioned in the main text. The DNA sequencing data of the scFv nucleotide sequence was deposited in the GenBank repository under the accession number OK642583.
